# Comparative Evaluation of the Effects of Legacy and New Generation Perfluoralkyl Substances (PFAS) on Thyroid Cells *In Vitro*

**DOI:** 10.3389/fendo.2022.915096

**Published:** 2022-06-23

**Authors:** Luca De Toni, Andrea Di Nisio, Maria Santa Rocca, Federica Pedrucci, Andrea Garolla, Stefano Dall’Acqua, Diego Guidolin, Alberto Ferlin, Carlo Foresta

**Affiliations:** ^1^ Department of Medicine, Unit of Andrology and Reproductive Medicine, University of Padova, Padova, Italy; ^2^ Unit of Andrology and Reproductive Medicine, University Hospital of Padova, Padova, Italy; ^3^ Department of Pharmaceutical Science, University of Padova, Padova, Italy; ^4^ Department of Neuroscience, Section of Anatomy, University of Padova, Padova, Italy

**Keywords:** endocrine disruptors, thyreocyte, iodide, PFOA, PFOS, C6O4

## Abstract

**Background:**

Per- and poly-fluorinated alkyl substances (PFAS) are environment-persitent emerging endocrine disrupting chemicals raising health concerns worldwide. Exposure to PFAS has been associated with the imbalance of thyroid hormones. However, available studies addressing the cell mechanism underlying thyroid disrupting feature of legacy PFAS, such as perfluoro-octanoic acid (PFOA), perfluoro-octane-sulfonic acid (PFOS), and the new generation substitutes, such as C6O4, are still lacking. In this study the potential disrupting effect of PFOA, PFOS, and C6O4 on a murine thyroid cell model was assessed.

**Methods:**

A rat FRTL-5 cell line was used as the normal thyroid follicular cell model. Cell iodide-uptake, induced by thyroid stimulating hormone (TSH), was used to assess the functional impact of PFAS exposure on cell function. Tetrazolium salt-based cell viability assay and merocyanine 540-based cell staining were used to address the possible involvement of cell toxicity and membrane biophysical properties on altered cell function. The possible direct interaction of PFAS with TSH-receptor (TSH-R) was investigated by computer-based molecular docking and analysis of molecular dynamics. Evaluation of intracellular cAMP levels and gene expression analysis were used to validate the direct impairment of TSH-R-mediated downstream events upon PFAS exposure.

**Results:**

Different from PFOS or C6O4, exposure to PFOA at a concentration ≥ 10 ng/mL was associated with significant impairment of the iodide uptake upon TSH stimulation (respectively: basal 100.0 ± 19.0%, CTRL + TSH 188.9 ± 7.8%, PFOA 10 ng/mL + TSH 120.4 ± 20.9%, p= 0.030 vs CTRL + TSH; PFOA 100 ng/mL + TSH 115,6 ± 12,3% p= 0.017 vs CTRL + TSH). No impairment of cell viability or membrane stability was observed. Computational analysis showed a possible direct differential interaction of C6O4, PFOA, and PFOS on a same binding site of the extracellular domain of TSH-R. Finally, exposure to PFOA was associated with a significant reduction of downstream intracellular cAMP levels and both sodium-iodide transporter and thyroperoxidase gene expression upon TSH-R stimulation.

**Conclusions:**

Our data suggest that legacy and new generation PFAS can differentially influence TSH dependent signaling pathways through the direct interaction with TSH-R.

## Introduction

In the last decades, great attention has been paid to the harmful effects of various chemicals on the environment and human health and how they interfere with hormonal function, collectively known as endocrine-disrupting chemicals (EDCs). Among EDCs, per- and poly-fluorinated alkyl substances (PFAS) are emerging chemicals raising health concerns worldwide (1). PFAS are a group of more than 4,700 anthropogenic chemicals (2). Among the family of perfluoroalkyl acids/polyfluoroalkyl acids, perfluoro-octanoic acid (PFOA) and perfluoro-octane sulfonic acid (PFOS) represent the two most diffused and most studied compounds. PFAS are used in a wide variety of consumer products and industrial applications because of their unique chemical and physical properties, including oil and water repellence, temperature and chemical resistance, and surfactant properties (3). For these reasons, PFAS have been used for several applications such as firefighting foams, non-stick metal coatings, food packaging, cosmetics, textiles, photography, chrome plating, pesticides, and pharmaceuticals. It is now recognized that within the large diversity of PFAS chemicals, some tend to accumulate in humans, animals, and the environment, adding to the total burden of other chemicals to which people are exposed and increasing the risk of health impacts.

Thyroid hormones play crucial roles in normal neurodevelopment of the fetus and child. Many chemicals can affect the control and homeostasis of thyroid hormones, eventually leading to various adverse health effects such as neurodevelopmental disorders. PFASs have been considered thyroid disrupting chemicals since the exposure to several PFASs was significantly associated with alteration of thyroid hormone (TH) balancing (4, 5). Thyroid hormones, namely triiodothyronine (T3) and thyroxine (T4), are produced by the thyroid and are finely regulated with a feedback mechanism by the thyroid stimulating hormone (TSH) that is produced in the pituitary gland. T3 and T4 play a crucial role in human metabolism, growth, and cell development and their dysregulation leads to an increased risk of developing cardiovascular diseases (6). Previous literature shows non-consistent results with positive, negative, and null associations between PFAS and T3 and T4, in the adult population (7–15) and in the youth age range (11, 16–18), although this variability has been mainly attributed to different degrees of PFAS exposure (19). A very recent study on 21,424 individuals aged 14–39 living in a highly contaminated area of Italy found no evidence of association between TSH and PFAS. However, some results suggested a possible inverse association of TSH with PFOA, and PFOS and perfluorohexane-sulfonic acid (PFHxS) among adult males (20).

The issue of PFAS as chemicals involved in the disruption effect of thyroid pathways was recently reviewed by Coperchini et al. (21). The research activity focused on the evaluation of the possible impact of PFAS exposure on the TSH-receptor (TSH-R)-mediated effects on available cell models of thyrocytes. However, their role as thyroid disruptors is still debated since *in vitro* experimental studies seem to confirm the detrimental effects of PFAS on thyroid cells (22, 23), from cytotoxicity and cell accumulation, to the interference with TPO function, iodine uptake, and TH synthesis. Moreover, in addition to *in vitro* studies, experiments on animal models have confirmed that both old and new generation PFAS act as thyroid disruptors (17, 24). Different mechanisms have been suggested to explain the thyroid disrupting feature of PFAS, including: i) impairment of iodine uptake of thyroid cells by competitive mechanisms and/or direct inhibition of the sodium/iodide symporter (NIS); ii) interference with thyroglobulin synthesis; iii) modification of thyroperoxidase (TPO) activity; iv) interference with feedback mechanisms or with thyroid hormone biological effects through disruption of the TH signaling pathway, deiodinase enzyme activity, or TH binding proteins (25).

On this basis, experimental models suggest that PFAS could exert a thyroid disrupting effect. However, epidemiological data in humans are not consistent in both adult and young subjects with varying degrees of exposure to these contaminants. In addition, most studies mainly focused on PFOS and PFOA, whereas adverse effects of their substitutes such as the acetic acid, 2,2-difluoro-2-[(2,2,4,5-tetrafluoro-5(trifluoromethoxy)-1,3-dioxolan-4-yl) oxy]-ammonium salt (1:1), known as C6O4, are still lacking. At present, only one study from Coperchini et al. showed no alteration of cell viability, ROS production, and cell proliferation in human thyroid cell lines exposed to C6O4, although specific endocrine-disrupting targets of thyroid cell function have not yet been evaluated (21). To this regard, we aimed to experimentally assess the potential disrupting effect of C6O4, PFOA, or PFOS on FRTL-5 normal rat thyroid follicular cell lines at multiple levels of investigation— from the functional impact of PFAS exposure on cell function, involvement of cell toxicity, and analysis of membrane biophysical properties to the computational modeling of the possible interaction of PFAS with TSH-R along with subsequent experimental validation.

## Methods

### Chemicals

Merocyanine 540 (MC540, cat#323756), thiazolyl blue tetrazolium bromide (MTT, cat#5655), nutrient mixture F-12 Ham (cat# F6636), hydrocortisone solution (cat# H6909), - bovine apo-transferrin (cat# T1428), somatostatin (cat# S1763), Gly-His-Lys acetate salt (cat# G7387), and insulin from bovine pancreas (cat# I6634) were all purchased by Merck Life Science S.r.l. (Milan, Italy). Native human thyroid stimulating hormones (TSH, cat# TSH-108H), were purchased by Creative BioMart Inc. (NY, USA). The Cyclic AMP Complete ELISA Kit (cat# ab133051), used for intracellular cAMP quantification, was purchased by Abcam (Cambridge, UK). The non-radioactive iodide Assay Kit (cat# 25659), used for intracellular iodide quantification, was purchased by Cayman Chemical (Michigan, USA). Perfluoro-octanoic acid and perfluoro-octane-sulfonic acid were purchased by Wellington Laboratories (Ontario, Canada). C6O4 was a kind gift from Solvay Industrial – HSE (Milan, Italy).

### Cell Cultures

The rat thyroid cell line FRTL-5 was a kind gift from Prof. Salvatore Ulisse (Department of Surgical Sciences. University of Rome “La Sapienza”, Rome, Italy). Cells were maintained under sterile conditions at 5% CO_2_ and 37°C in Coon′s modified Ham′s F12 medium with 2mM Glutamine, 10μg/ml Insulin, 10nM Hydrocortisone, 5μg/ml Transferrin, 10ng/ml gly-his-lys acetate, 10ng/ml somatostatin, 5% Foetal Bovine Serum (FBS), and 1 mU/ml TSH as previously described (26). Sub-confluent cultures (70-80% confluence) were detached with 0.25% trypsin-EDTA solution and seeded at 3-4×10,000 cells/cm^2^ density. In stimulation experiments, sub-confluent cells underwent overnight starving in culture medium lacking TSH. Cells were then exposed to PFAS for 24 hours and then evaluated as described below.

Accumulation of PFAS levels in FRTL-5 cell culture were measured through reversed-phase (RP) liquid chromatography coupled with triple quadrupole mass spectrometry (LC-MS/MS) as previously described (27)

### Cell Toxicity and Membrane Fluidity

The possible inhibition of cell growth by PFAS was evaluated by MTT assay as previously described (28). Briefly, 3-8×10^3^ cells/well were seeded in 96-well microplates in 100 μL culture medium. After 24 hours of culture, cells were exposed to PFAS diluted in complete medium at concentration ranging from 0.1 to 100 ng/mL as indicated below. Triplicate cultures were established for each treatment. After 24 hours of exposure, cells were incubated for 5 hours with MTT at the final concentration of 0.5 mg/mL and then lysed with a solution of 1% sodium dodecylsulfate (SDS) in HCl 0.01 M. After an overnight treatment, the absorbance of each well at 570 nm was measured by a Bio-Rad 680 microplate reader and normalized on controls lacking PFAS exposure.

Cell membrane fluidity was assessed by the bilayer sensitive probe Merocyanin 540 (MC540), as previously described (29–31). Briefly, DMSO-stock solution of MC540 was diluted in cell suspension at the final concentration of 4 μM and incubated for 15 minutes at 37°C in the dark. Cells were finally analyzed at FACScan flow cytometer (Becton Dickinson, Milan, Italy). Mean cell fluorescence intensity was normalized on controls lacking PFAS exposure.

### Computer-Based Molecular Docking and Molecular Dynamics Analysis

The possible docking of PFAS to TSH receptor (TSH-R) was investigated through a computational approach. The structure of the extracellular domain of the human (TSH-R) is available as deposited 3D structure in the Protein Data Bank (PDB code 2XWT). The computational docking analyses were carried out by considering only this domain. Once extracted from crystallography template, TSH-R was subjected to energy minimization by Yasara Energy Minimization Server (YASARA Energy Minimization Server) to obtain an estimate of its unliganded configuration. As there are no experimental models of TSH-R in complex with TSH, and it was estimated using the experimental models of the 2 TSH subunits and taking the FSH-FSHR complex as a template, whose structure was experimentally obtained (PDB code: 1XWD).

The possibile binding of PFAS to the TSH-R extracellular domain was evaluated by the Autodock Vina algorithm (32) implemented in the UCSF Chimera 1.12 (https://www.cgl.ucsf.edu/chimera/) molecular modeling software (33).

The molecular dynamics procedure, based on the method by Kuriata et al. (34) and available as a web server (https://biocomp.chem.uw.edu.pl/CABSflex2), was used to evaluate the conformations the receptor can acquire after binding with PFAS and to compare them with those of the free receptor.

### RNA Isolation, cDNA Synthesis, and Real-Time PCR

Total RNA was extracted from FRLT5 cell line stimulated with increasing concentrations of PFOA, PFOS, C6O4 using the RNeasy Mini Kit (Qiagen, Hilden, Germany). Dnase treatment was performed using Ambion^®^ TURBO DNA-free™ Kit (Thermo Fisher Scientific, Carlsbad, CA, USA) according to the manufacturer’s instruction. RNA purity and concentration was assessed by NanoDrop ND-1000 (NanoDrop Technologies, Wilmington, DE, USA).

cDNA was synthesized from 500 ng of total RNA using SuperScript III (Invitrogen, Carlsbad, CA, USA) and random hexamers. Real Time PCR were performed in a 20 µl final volume containing 20 ng of cDNA, 1X Power SYBR Green PCR Master Mix (Applied Biosystem, Foster City, CA, USA), and a mix of forward and reverse primers (1 mmol/l each). The following primers were used: Nis forward 5′-TCCTCACAGGCCGTATCTCA-3′ and reverse 5′-GAAGGAACCCTGGAGGACAC-3′, Tpo forward 5′-GCATGTATCATTGGGAAGCA-3′ and reverse 5′-CGGTGTTGTCACAGATGACC-3′, Tshr forward 5′- TCATTGCCTCTGTAGACCTG-3′ and reverse 5′- TGATAACTCACTGGCGAAA-3′. Mouse Beta actin was used as a reference gene: forward 5′- GGCACCACACTTTCTACAATG-3′ and reverse 5′- TGGCTGGGGTGTTGAAGGT-3′. StepOne plus (Applied Biosystems, Foster City, CA, USA) thermocycler was used for Real Time PCR and relative quantification was performed using Delta Delta Ct (2−ΔΔCt) method.

### Quantification of Intracellular cAMP and Iodide Uptake

TSH-induced cAMP production in FRTL-5 cells was assessed as previously described (35). Upon the achievement of 80% confluence in complete medium, cells were starved for 72h in medium without TSH. Subsequently, cells were incubated for another 24 hours in complete medium with PFAS at concentration ranging from 0.1 to 100 ng/mL. In control samples, PFAS were omitted. Cells were then harvested by scraping in ice-cold phosphate-buffered saline (PBS) and cAMP was quantified with Competitive Cyclic AMP ELISA Kit according to the manufacturer’s instructions.

Non radioactive cell iodide uptake was assessed in FRTL-5 cells as previously described (36). Briefly, cells were seeded in 96-well plates and grown up to 60% confluence in complete medium. Cells were then starved in growth medium without TSH for 72 hours, followed by 24 hours exposure to PFAS, at a concentration ranging fron 0.1 to 100 ng/mL and stimulation with TSH 1 mU/ml for another 48h. After the treatments, cells were washed twice with 1 ml HEPES-buffered modified Hank’s balanced salt solution (HBSS) (137 mM NaCl, 5.4 mM KCl, 1.3 mM CaCl2, 0.4 mM MgSO4, 0.5 mM Na2HPO4, 0.44 mM KH2PO4, 5.55 mM glucose, 10 mM HEPES, pH 7.3) and incubated for 60 min at 37°C modified HBSS containing 1 mM NaI. Cells were then washed twice with ice-cold modified Hank’s Balanced-Salt Solution (HBSS) and then assessed for iodide concentration a with non-radioactive iodide Assay Kit according to manufacturer’s instruction. Reference inhibition of iodide uptake was obtained by incubation with 50 μM sodium thiocyanate (NaSCN).

All experiments were performed in triplicate. Data were normalized on control conditions free from PFAS exposure and TSH stimulation.

### Statistical Analysis

Statistical analysis of the data was conducted with SPSS 21.0 for Windows (SPSS, Chicago, IL, USA). The comparison between the two groups of data, obtained from western blot analysis and cytochemical staining, were determined by paired two-tailed Student’s t-test after acceptance of normal distribution of the data with the Kolmogorov–Smirnov test. One-way ANOVA with Bonferroni correction was used for the comparison of more than 2 groups of data. Values of p<0.05 were considered as statistically significant.

## Results

### Exposure to Legacy Perfluoroalkyl-Substance PFOA Alters TSH-Mediated Cell Iodide Uptake Without the Involvement of Cell Viability or Membrane Biophysical Properties

**Figure 1:**
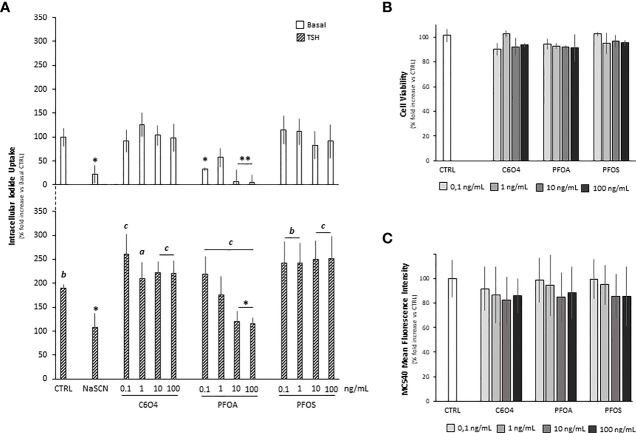
**(A)** Evaluation of cell iodide uptake, at basal condition (Basal)and upon stimulation with 1 mU/ml thyroid stimulating hormone (TSH), in FRTL-5 normal rat thyrocyte cell line exposed for 24 hours to acetic acid, 2,2-difluoro-2-[(2,2,4,5-tetrafluoro-5(trifluoromethoxy)-1,3-dioxolan-4-yl)oxy]-ammonium salt (1:1) (C6O4), perfluoro-octanoic acid (PFOA) perfluoro-octane-sulfonic acid (PFOS) at concentration ranging from 0.1 to 100 ng/mL. Reference inhibition of iodide uptake was obtained by incubation with 50 μM sodium thiocyanate (NaSCN). Experiments were performed in triplicate and normalized as percentage fold increase on basal control laking TSH stimulus and exposure to perfluoroalkyl substances (CTRL). Data are reported as mean values ± standard deviation. Significance: *P < 0.05, **P < 0.01 vs corresponding CTRL; a=p < 0.05, b=p < 0.01 and c=p < 0.001 vs corresponding basal). Evaluation of FRTL-5 cell viability **(B)** and cell mebrane fluidity **(C)** upon exposure to C6O4, PFOA or PFOS exposed for 24 hours at concentration ranging 0.1 to 100 ng/mL. In CTRL, exposure was omitted. Cell viability was evaluated by MTT assay and membrane fluidity by merocyanine-540 staining as described in methods. Experiments were performed in triplicate and normalized as percentage fold increase on CTRL. Data are reported as mean values ± standard deviation.

In order to evaluate whether the exposure to PFAS was associated with altered thyrocyte cell function, the impact on TSH-dependent iodide uptake was firstly evaluated. To this end, starved FRTL-5 were exposed for 24 hours to C6O4, PFOA, or PFOS at a concentration ranging from 0.1 ng/mL to 100 ng/mL and then stimulated with human TSH 1 mU/ml (**Figure 1A**). Sodium thyocyanate (NaSCN), a known blocker of NIS, was used as reference downstream inhibitor of iodide uptake (37).

At basal conditions, in absence of TSH stimulation, compared to the unexposed control (100.0 ± 19.0%), cell iodide uptake was significantly reduced upon NaSCN treatment (22,2 ± 18,3%, p=0.0036). In addition, differentl from C6O4 and PFOS for which no major effects were observed, a significant reduction of basal iodide uptake was observed in cells exposed to PFOA at the concentration of 0.1 ng/mL (p= 0.0366) and at a concentration equal or greater than 10 ng/mL (respectively PFOA 10 ng/mL 7.4 ± 23.6%, p=0.0014 and PFOA 100 ng/mL 5.6 ± 15%, p=0.0012).

Stimulation with TSH was associated with a significant increase of cell iodide uptake in all tested conditions [respectively, versus corresponding basal: CTRL+TSH 188.9 ± 7.9%, p= 0.0025, NaSCN+TSH 107.4 ± 29.0% p=0.045; for cells exposed to C6O4 at any concentration *F*(1, 20)=18.56 and p<0.001, for cells exposed to PFOA at any concentration *F*(1, 20)=36.15 and p<0.001 and for cells exposed to PFOS at any concentration *F*(1, 20)=30.94 and p<0.001]. However, a peculiar trend emerged from the comparison with the CTRL condition stimulated with TSH. As expected, the treatment with NaSCN significantly reduced cell iodide uptake (p=0.045). Interestingly, no significant alteration of cell iodide uptake upon TSH stimulation was observed for cells exposed to C6O4 or PFOS (all p>0.05 *vs* CTRL+TSH), while exposure to PFOA at a concentration equal or greater than 10 ng/mL was associated with a significant reduction of this parameter (respectively: PFOA 10 ng/mL + TSH 120.4 ± 20.9%, p= 0.030, PFOA 100 ng/mL + TSH 115,6 ± 12,3% p= 0.017), suggesting a major impact of this perfluorinated compound on TSH-mediated function in the thyrocyte model.

In order to rule out a major involvement of cell viability in the observed altered function of thryrocyte upon exposure to PFOA, an MTT-based cytotoxicity test was performed (**Figure 1B**). Compared to unexposed control, the exposure to C6O4, PFOA, or PFOS for 24 hours at a concentration ranging from 0.1 to 100 ng/mL was associated with non significant variation of cell viability at any tested condition.

Based on previous reports, major impairment of cell function associated with PFAS exposure may rely on the alteration of membrane bilayer stability (30, 38, 39). Accordingly, we evaluated whether the observed impaired thryrocyte function upon exposure to PFOA was associated with the membrane’s altered biophysical properties, such as fluidity. To this end, we used the bilayer fluidity-sensitive probe Merocyanin 540 (MC540; **Figure 1C**). The exposure to C6O4, PFOA, or PFOS for 24 hours at a concentration ranging from 0.1 to 100 ng/mL was associated with non significant variation of the mean fluorescence intensity of MC540 staining, a proxy of membrane fluidity, compared to unexposed control. Accordingly, no significant cell accumulation was detected for any PFAS at LC-MS/MS analysis (data not shown) measured using our previously published method (27).

### Computer-Based Molecular Docking and Molecular Dynamics Analysis Shows the Possible Interference of Legacy PFAS on the TSH/TSH-R Interaction

**Figure 2:**
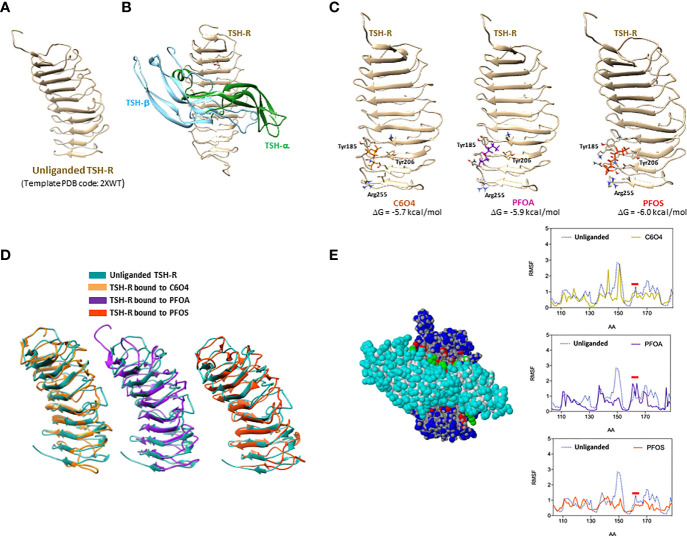
Computer-based Molecular Docking and Molecular Dynamics Analysis of the possible docking of 2,2-difluoro-2-((2,2,4,5-tetrafluoro-5(trifluoromethoxy)-1,3-dioxolan-4-yl)oxy)-ammonium salt (1:1) (C6O4), perfluoro-octanoic acid (PFOA) or perfluoro-octane sulphonic acid (PFOS), to thyroid stimulating hormone-receptor (TSH-R), whose extracellular domain is shown in **(A)**. Representative model of thyroid stimulating hormone (TSH) eterodimeric chain (TSH-α and TSH-β) bound to TSH-R is reported in **(B)**. Possible binding sites of C6O4, PFOA or PFOS to TSH-R are reported in **(C)**, with the the corresponding free Gibbs energy variations (ΔG) of the complex formation and aminoacids involved in the binding. Representative images of the best conformers of unliganded receptor (blue) overimposed with the receptor bound to C6O4 (ocra), PFOA (purple) or PFOS (orange), are shown in **(D)**. The interaction interface between TSH and the extracellular domain of TSH-R and the the root-mean-square deviation (RMSD) profile of the LEU100-GLN170 domain of the receptor, mainly involved in the binding to TSH, of unliganded receptor and the receptor bound to C6O4, PFOA, or PFOS, are shown in **(E)**.

In order to address the possible direct effect of PFAS exposure on TSH-mediated signaling, the binding of these compounds to TSH-R was investigated through a computational approach, focusing on the extracellular domain of the receptor, whose structure is available in the Protein Data Bank (PDB code 2XWT, **Figure 2A**). The consistency between rat and human TSH-R was assumed on the basis of the responsiveness of the murine model to human TSH. Importantly, the binding mode of TSH to TSH-R (**Figure 2B**) was estimated using the FSH-FSHR complex as template (PDB code: 1XWD).

On this basis, the possible binding of C6O4, PFOA, and PFOS as representative models of new generation and legacy PFAS, was assessed on the unliganded TSH-R structure. Interestingly, a binding site was observed for all the considered PFAS in the same domain of the receptor involving the peptide sequence TYR-185-ARG-255 (**Figure 2C**). In addition, the binding models of the three molecules showed camparable stability, being associated with similar free Gibbs energy variations estimates (-5.7 kcal/mol for C6O4, -5.9 kcal/mol for PFOA and -6.0 kcal/mol for PFOS).

Molecular dynamics procedures allowed the evaluation of the differential conformations acquired by the receptor upon the binding of the three PFAS molecules, compared to the unliganded receptor. **Figure 2D** shows the best conformers of unliganded receptor and of the receptor bound to C6O4, PFOA, and PFOS, highlighting major differences of the two equilibrium configurations. It can therefore, be hypothesized that the binding of the two legacy and the new-generation PFAS to TSH-R can differentially modify the affinity for TSH, resulting in major alteration of the downstream signal transduction. This hypothesis was further investigated by the analysis of the estimated flexibility of the extracellular domain of the receptor in the different binding conditions. **Figure 2E** shows the interaction interface between TSH and the extracellular domain of TSH-R, where the aminoacid residues mainly involved of the receptor are in the LEU100-GLN170 domain of the receptor. The root-mean-square deviation (RMSD) profile of the atomic positions in this domain for the unliganded receptor compared to that of the TSH-R bound to C6O4, PFOA, or PFOS (**Figure 2E**), depicts a different spectrum of flexibility. In particular, focusing on the amino acid region of TSH-R around ASP160, previously shown to highly involved in the activation by TSH (40), shows that the modelled binding of PFOA was associated with an increase of the backbone flexibility, compared to both the unliganded receptor and the receptor bound to C6O4 or PFOS.

### Exposure to PFOA Alters Main Downstream Events of the TSH-R-Mediated Signaling Pathway

In order to provide a mechanistic link between the observed altered thyrocyte cell function and the possible differential interference of the PFAS in the interaction between TSH and TSH-R, the analysis of main downstream events of the TSH-R mediated signaling pathway was performed.

**Figure 3:**
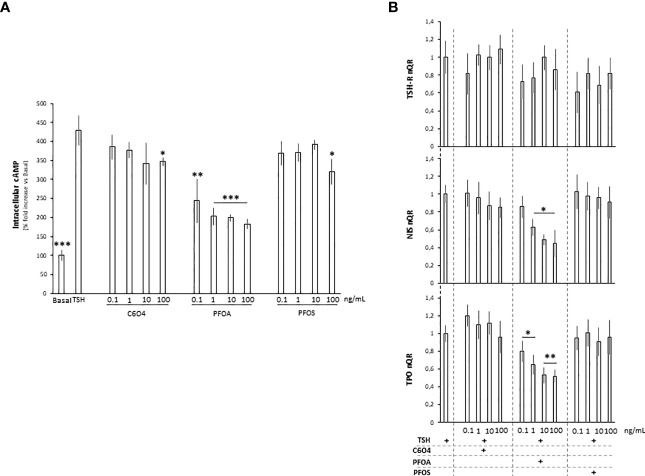
**(A)** Evaluation of intracellular cAMP levels in FRTL-5 normal rat thyrocyte cell line at basal condition, upon stimulation with 1 mU/ml thyroid stimulating hormone (TSH) or after the exposure for 24 hours to acetic acid, 2,2-difluoro-2-((2,2,4,5-tetrafluoro-5(trifluoromethoxy)-1,3-dioxolan-4-yl)oxy)-ammonium salt (1:1) (C6O4), perfluoro-octanoic acid (PFOA) perfluoro-octane-sulfonic acid (PFOS), at concentration ranging from 0.1 to 100 ng/mL, followed by stimulation with TSH. Experiments were performed in triplicate and normalized as percentage fold increase on basal control lacking TSH stimulus and exposure to perfluoroalkyl substances (CTRL). Data are reported as mean values ± standard deviation Significance: *P < 0.05, **P < 0.01, ***P < 0.001 *vs* unexposed cells stimulated with TSH. **(B)** Evaluation of TSH-receptor (TSH-R), sodium-iodide symporter (NIS) and thyroperoxidase (TPO) gene expression FRTL-5 cell at different combinations of exposure to C6O4, PFOA and PFOS, at concentration ranging from 0.1 to 100 ng/mL, and upon stimulation with TSH. Experiments were performed in triplicate and normalized on cell stimulated with TSH. Significance: *P < 0.05, **P < 0.01, ***P < 0.001 vs TSH.

TSH-R belongs to the family of G-protein-coupled receptors, whose activation by TSH triggers adenylate cyclase (AC) activation and, in turn, increases intracellular levels of cyclic adenosine monophosphate (cAMP) to promote the pathways transduced by protein kinase A (PKA) (41). On this basis, the possible impact of PFAS on intracellular cAMP levels upon TSH stimulation was investigated (**Figure 3A**). As expected, stimulation with TSH was associated with a massive increase of intracellular cAMP levels compared to unstimulated basal conditions (respectively: basal 100.0 ± 14,1% vs TSH 428.9 ± 38.4%, p= 0.0002). Compared to unexposed rat thyrocytes stimulated with TSH, cells exposed for 24 hours to C6O4 or PFOS at concentration ranging from 0.1 to 100 ng/mL and stimulated with TSH, showed a significant reduction of intracellular cAMP levels only at the highest concentration tested (C6O4 100 ng/mL 347.2 ± 11.1%, p=0.023; PFOS 100 ng/mL 320.0 ± 33.3, P=0.020). Differently, exposure to PFOA was associated with a significant reduction of intracellular cAMP levels upon TSH stimulation at all concentrations tested (PFOA 0.1 ng/mL 244,1 ± 57,6%, p= 0.01; PFOA 1 ng/mL, p= 0.001; PFOA 10 ng/mL, p= 198.9 ± 8.2%, p=0.0005; PFOA 100 ng/mL 183.0 ± 12.2, p=0.0005).

The direct phosphorylation of the cAMP-responsive element binding protein (CREB) represents a key event in TSH-receptor-related signaling, driving the expression of the downstream genes such as NIS and thyroperoxidase (TPO) (42, 43). Accordingly, the possible effect of PFAS exposure on the downstream gene expression of NIS and TPO upon TSH stimulation was evaluated (**Figure 3B**). In spite of non- significant effects of either TSH or PFAS and TSH on TSH-R gene expression, exposure to PFOA for 24 hours was associated with a significant reduction of both NIS and TPO gene expression upon TSH stimulation, at all the tested concentrations. Differently, exposure to C6O4 or PFOS exerted no major alteration of the gene pattern.

## Discussion

This is the first study to compare the *in vitro* adverse effects of legacy and new-generation PFAS on a cell model of thyrocyte function and TSH-R mediated pathways. In particular, we focused on the most peculiar markers of thyrocyte function, such as iodide uptake, for which a disrupting effect was observed, essentially for PFOA. Importantly, this effect is not due to non-specific endpoints such as cell viability or membrane biophysical properties in the range of concentrations from 0.1 to 100 ng/mL. The lack of a cytotoxic effect on thyroid cells is in agreement with a recent study on the same molecules from Coperchini et al. (21). Conversely, the absence of significant impairment of membrane fluidity, evaluated by merocyanine 540 staining, represents a novel result, particularly compared with previous studies in which PFAS exposure was associated with an increased membrane fluidity in different cell models, such as dopaminergic neurons (27), sperm (38). or platelets (39). The different behavior of thyroid cells could be explained by the different membrane composition and plasticity, leading to a more rigid membrane in physiological conditions and less prone to modifications from external insults (44). The fact that the exposure to PFOA, but not C6O4 or PFOS, negatively affected iodide uptake even at the lowest concentration tested, led us to investigate the potential interaction of the three molecules with TSH-R by a computer-based approach. It should be noted that the amino acid residues primarily inolved in TSH-R activation by TSH have been previously identified by Sanders et al. through a site-mutation approach (40). Interestingly, among the 41 experimental genetic variants tested, only those related to amino acids GLU107, ASP160, ASP232, and TYR279 were associated with a significant impairment of early TSH-R-mediated intracellular cAMP increase. We found that both three PFAS show a possible binding activity on a shared site of the extracellular domain of TSH-R. Despite the fact that none of the aforementioned amino acids critically involved in TSH-R activation were committed in the binding to PFAS, molecular dynamic analysis showed a differential pattern of the receptor’s backbone flexibility associated, in particular, at the ASP160 site within the putative receptor domain involved in the interaction with TSH. This pattern of distal inhibition is not novel. In fact, with an analog site mutation approach, the blocking autoantibody K1-70 was shown to bind the TSH-R closed to the N-terminus with minimal overlap on TSH-binding domain (45). Accordingly, this evidence can possibly explain the differential effects of the three PFAS tested on TSH-R mediated cell function. Major uncertainty may be derived from the modelling on the human receptors domain while *in vitro* results are from rat cells. However, the consistency between rat and human TSH-R can be assumed on the basis of the responsiveness of the murine model to human TSH.

Results from previous studies on humans reported that significant concentrations of PFOA and PFOS can be detected in thyroid gland surgical specimens (46) and suggest that PFAS could be potential endocrine disruptors, interfering with the physiological mechanisms of hormone regulation (47), including thyroid hormones. The biosynthesis of thyroid hormones and TSH is finely regulated by a negative feedback mechanism involving the hypothalamic–pituitary–thyroid axis, with an increase in thyroid hormones leading to a decrease in TSH and vice versa (48). However, available *in vitro* studies show that legacy PFAS compounds exert a cytotoxic effect on the thyroid only at very high concentrations (22). In a context in which studies about cell effects of C6O4 exposure are still scarce (21, 29, 49, 50), only one to date has evaluated the impact of this new generation PFAS on thyroid cells (21), showing a lower toxicity of C6O4 compared with legacy PFAS, mainly PFOA and PFOS, and largely overlapping with our results. In fact, we confirm here the lack of a pro-apoptotic or genotoxic effect of C6O4 on thyreocyetes and add novel data about a possible specific effect on the endocrine pathways related to thyroid function, showing that among long-chain PFAS, PFOA displays a more severe disrupting effect as compared to PFOS and C6O4. Taken together, these data indicate that PFAS, even if structurally related, show different toxicity profiles also depending on the target cell considered.

A remaining uncertainty of the study is related to the concentrations of C6O4 which were tested in this study, being in the same dose range reported for blood serum levels of PFOA and PFOS in population exposure studies. This is clearly a provisional setting in the absence of serum levels of C6O4 in general public or industry workers. Also, we tested these compounds independently but in real life context, a cocktail effect in which humans are exposed to a mixture of PFAS and also other endocrine disruptors is much more realistic. To this regard, a very recent study has evaluated a mixture-centered risk assessment strategy, integrating epidemiological and experimental evidence. The mixture tested included also PFAS and confirmed a detrimental effect on thyroid-related pathways (51). Finally, the results of the present study would require confirmation in human cell types and in animal models before extrapolating conclusive data on a human safety profile.

## Conclusion

Our data suggest that legacy and new generation PFAS can influence TSH dependent signaling pathways through a possible differential direct interaction with TSH-R, according to the structural moiety of the molecule. Further study in human cell and in animal models are requested to address the consistency and reliability of these preliminary data

## Data Availability Statement

The raw data supporting the conclusions of this article will be made available by the authors, without undue reservation.

## Ethics Statement

The study involved the use of a commercially available rat cell model, requiring no approval by the local Ethics Committee

## Author Contributions

LDT and ADN wrote the first draft of the manuscript, conducted the data analysis and interpretation and contributed to the collection and standardization of the data. FP performed *in vitro* experiments. MSR performed gene expression analysis. DG performed computer-based molecular docking and molecular dynamics analysis. SD’A performed LC-MS analysis. AG, AF, and CF contributed to the discussion, conducted the research, and collected the data. LDT, ADN, and CF designed the study and revised the manuscript. All authors read and approved the final manuscript.

## Funding

The study was funded by a grant from Solvay Specialty Polymers Italy S.p.A. The funder had no role in the design and conduct of the study; collection, management, analysis, and interpretation of the data, or approval of the manuscript; and decision to submit the manuscript for publication.

## Conflict of Interest

The authors declare that the research was conducted in the absence of any commercial or financial relationships that could be construed as a potential conflict of interest.

## Publisher’s Note

All claims expressed in this article are solely those of the authors and do not necessarily represent those of their affiliated organizations, or those of the publisher, the editors and the reviewers. Any product that may be evaluated in this article, or claim that may be made by its manufacturer, is not guaranteed or endorsed by the publisher.
